# Using Two Bursts of MIDUS to Inform Daily Positive Experience and Physical Health Symptom Associations

**DOI:** 10.1093/geroni/igaf122.826

**Published:** 2025-12-31

**Authors:** Dakota Witzel, Maria Kurth, Shelbie Turner, Robert Stawski

**Affiliations:** South Dakota State University, Brookings, South Dakota, United States; The Pennsylvania State University, University Park, Pennsylvania, United States; Weill Cornell Medicine, New York, New York, United States; Utah State University, Logan, Utah, United States

## Abstract

Positive experiences are buffers for long-term health outcomes such as mortality and daily objective health indicators such as cortisol and inflammation. However, less is known about the implications of positive experiences on proximal (i.e., same day) subjective physical health symptoms. Using two bursts of data from the National Study of Daily Experiences (N = 1,327, Magewave2∼56, Nobs=7,966), we examined whether the number and type of daily positive experience was associated with same day physical health symptoms (M = 19), as well as 10-year changes therein. Further, we explored age-related resilience of positive experiences by considering age as a moderator. In both Wave 2 (year: 2005) and Wave 3 (year: 2015), participants completed eight days of end-of-day telephone interviews reporting on their daily experiences including daily positive experiences (interpersonal interactions, at work, and at home) and physical health symptoms (e.g., headache, muscle soreness, diarrhea). Both the number and type of positive experiences were significantly related to the number of physical health symptoms reported in a day (ps < .05). Days when adults reported either a positive interpersonal interaction, work, or home experiences was associated with more physical health symptoms compared to when no positive experience was reported. The effect of positive interpersonal interactions was statistically significant for middle aged adults (estimate=.12, p< .001) but not older adults (estimate=.01 p=.56). Discussion will include how daily positive experiences may be an indicator of an active life, thus resulting in more daily symptoms such as aches and highlight potential bidirectional effects.

